# NSvc4 Encoded by Rice Stripe Virus Targets Host Chloroplasts to Suppress Chloroplast-Mediated Defense

**DOI:** 10.3390/v14010036

**Published:** 2021-12-24

**Authors:** Zongdi Li, Chenyang Li, Shuai Fu, Yu Liu, Yi Xu, Jianxiang Wu, Yaqin Wang, Xueping Zhou

**Affiliations:** 1 State Key Laboratory of Rice Biology, Institute of Biotechnology, Zhejiang University, Hangzhou 310058, China; 11816056@zju.edu.cn (Z.L.); applne@hotmail.com (C.L.); fushuai@zju.edu.cn (S.F.); 11716019@zju.edu.cn (Y.L.); xuyiqdpd@njau.edu.cn (Y.X.); 2 State Key Laboratory for Biology of Plant Diseases and Insect Pests, Institute of Plant Protection, Chinese Academy of Agricultural Sciences, Beijing 100193, China; 3 Department of Plant Pathology, Nanjing Agricultural University, Nanjing 210095, China

**Keywords:** rice stripe virus, movement protein, chloroplast, defense, virus–chloroplast interaction, NbGAPDH-A, NbPsbQ1

## Abstract

Our previous research found that NSvc4, the movement protein of rice stripe virus (RSV), could localize to the actin filaments, endoplasmic reticulum, plasmodesmata, and chloroplast, but the roles of NSvc4 played in the chloroplast were opaque. Here, we confirm the accumulation of NSvc4 in the chloroplasts and the N-terminal 1–73 amino acids of NSvc4 are sufficient to localize to chloroplasts. We provide evidence to show that chloroplast-localized NSvc4 can impair the chloroplast-mediated immunity. Expressing NSvc4 in *Nicotiana benthamiana* leaves results in the decreased expression of defense-related genes *NbPR1*, *NbPR2*, and *NbWRKY12* and the inhibition of chloroplast-derived ROS production. In addition, generation of an infectious clone of potato virus X (PVX) carrying NSvc4 facilitates PVX infection in *N. benthamiana* plants. Moreover, we identify two chloroplast-related host factors, named NbGAPDH-A and NbPsbQ1, both of which can interact with NSvc4. Knockdown of *NbGAPDH-A* or *NbPsbQ1* can both promote RSV infection. Our results decipher a detailed function of NSvc4 in the chloroplast.

## 1. Introduction

Plants are constantly threatened by various forms of stresses throughout their life, from the extreme external environment to microbial pathogens [1]. To survive from these challenges, plants have evolved a set of complex immune systems, including pattern-triggered immunity (PTI) and effector-triggered immunity (ETI) [2]. The former pattern is induced by pathogen-derived molecules recognized by plasma membrane (PM)-localized receptors. The output is usually associated with reactive oxygen species (ROS) burst, callose deposition, downstream defense-related genes expression, and plant hormones synthesis [3]. ETI occurs when plant resistance genes (R genes) recognize the pathogen effectors, resulting in the hypersensitive response activity (HR) at the infection sites [4].

Chloroplast is an indispensable organelle for plants; its function is more than capturing light and converting it to chemical energy. It also plays a crucial role in the arms race between pathogens and plants [5,6,7]. ROS are one of the most effective molecules for plant immunity, which is a byproduct of normal cell metabolism in plants and can also be produced in the chloroplasts during photosynthesis [8]. The production of ROS can not only inhibit the pathogen colonization but also acts as the defense signal for chloroplast to communicate with the nucleus and subsequently activates the expression of defense-related genes and synthesis of defense hormones in the chloroplast, such as salicylic acid (SA) [9]. Since chloroplast is of great significance to plant immunity, pathogens have evolved many strategies to break through the obstacles set by plants in order to achieve successful infection. They encode effectors to target chloroplasts to weaken the chloroplast-dependent defense [10], which is widely activated against pathogens from different kingdoms.

There is an increasing number of reports about virus–chloroplast interactions [11]. Virus infection affects the structure and function of chloroplasts. For instance, potato virus Y (PVY) infection causes the reduced number and size of chloroplast [12]. The infection of potato mop-top virus (PMTV) in *N. benthamiana* plants leads to chloroplast abnormalities, including larger starch grains, large cytoplasmic inclusion, and terminal extension [13]. Chloroplast is an ideal place for virus proliferation, because the chloroplast envelope can protect viral RNA from host RNAi-mediated defense [14]. For example, the viral genomic RNA and coat protein (CP) of PMTV are associated with chloroplasts in PMTV-infected *N. benthamiana* plants, suggesting that chloroplasts might be the sites of virus replication [13]. The interaction between viral RNAs of bamboo mosaic virus (BaMV) and the chloroplast phosphoglycerate kinase (chl-PGK) is essential for the replication of BaMV [15]. Moreover, chloroplast-targeting viral proteins can suppress chloroplast-mediated defense. C4 protein of tomato yellow leaf curl virus (TYLCV) can be redirected from PM to chloroplast to inhibit SA-dependent immunity [16].

RSV is a single-stranded negative RNA (ssRNA-) virus, belonging to the genus *Tenuivirus*. RSV is transmitted by small brown planthopper *Laodelphax striatellus* in a persistent-propagative manner and causes typical chlorotic stripe symptoms on rice, leading to huge yield losses in East Asian rice producing areas [17]. The RSV genome consists of four RNAs and encodes seven proteins by using both negative-sense and ambisense-coding strategies, namely RNA-dependent RNA polymerase (RdRp), NS2, NSvc2 (glycoprotein), NS3 (gene silencing suppressor), NSvc3 (CP), NS4 (disease-specific protein, SP), and NSvc4 (movement protein, MP) [18].

NSvc4 belongs to the virus movement protein “30 K” superfamily and is responsible for virus movement [19]. Similar to other MPs from a wide range of genera of viruses, NSvc4 could localize to the plasmodesmata (PD) and is capable of complementing the cell-to-cell movement of a movement-deficient potato virus X (PVX) [19]. Our previous research found that NSvc4 could also localize to actin filaments, endoplasmic reticulum, and chloroplast [20], but the functional role of NSvc4 in the chloroplast need further investigation. In our present study, we confirmed that NSvc4 accumulated in *N. benthamianan* chloroplasts during RSV infection to downregulate chloroplast-mediated immunity to support virus infection. We also identified two host chloroplast-related factors interacting with NSvc4, NbGAPDH-A and NbPsbQ1, whose downregulation promotes RSV infection.

## 2. Materials and Methods

### 2.1. Plant Growth Condition and Virus Inoculation

The growth condition for *N. benthamiana* plants was set at 25 °C, 60% relative humidity, and the photoperiod was 16 h in light and 8 h in dark. RSV mechanical inoculation was described previously [21].

### 2.2. Plasmid Construction

Total RNA was extracted from wild-type and RSV-infected *N. benthamiana* plants by using TRIzol reagent (Invitrogen, Carlsbad, CA, USA), as described previously [22]. ReverTra Ace qPCR RT Master Mix with gDNA Remover Kit (TOYOBO, Osaka, Japan) was used to obtain cDNA following the instructions. Genes from *N. benthamiana* and RSV were cloned by PCR amplification with specific primer pairs and fused into corresponding protein expressing vectors. As for the construction of in planta expression vectors, the pCV vector was digested by *BamH*I and *Sac*I, the pGD-FLAG vector was digested by *Sal*I, and NSvc4, mNSvc4 were fused into the vectors by using the ClonExpress II One Step Kit (Vazyme, Nanjing, China) according to the instructions. For subcellular localization assays, the pGD-eGFP and pGD-mCherry vectors were digested by *BamH*I, NSvc4, and its mutants, as well as host factors NbGAPDH-A and NbPsbQ1 fused into vectors by using the ClonExpress II One Step Kit. For BiFC assays, NSvc4 and GUS were fused into vectors carrying the N-terminal halves of YFP by T4 DNA ligase (Thermo Fisher, Waltham, MA, USA), and NbGAPDH-A and NbPsbQ1 were fused into vectors carrying C-terminal halves of YFP by using the same methods. For TRV-based VIGS, the TRV RNA2 vector was digested with *Xba*I and *Xho*I, and 200–300-bp fragments of NbGAPDH-A and NbPsbQ1 were constructed into the TRV RNA2 vector. The sequences of all primers used in this study are provided in Table S1.

### 2.3. Transient Expression in N. benthamiana

Transient expression assays were performed as described previously [16]. Briefly, the *Agrobacterium tumefaciens* strains EHA105 harboring the corresponding expressing vectors and GV3101 strains carrying PVX vectors were cultured in YEP liquid medium with the appropriate antibiotics at 28 °C overnight. Bacterial cultures were centrifuged at 12,000× *g* for 1 min and resuspended in the infiltration buffer (10-mM MgCl_2_, 10-mM MES, pH 5.6, and 150-μM acetosyringone) to an OD600 = 0.6–0.8. Before infiltration, the bacterial suspensions were kept at room temperature for 2 h. Five-leaf *N. benthamiana* plants were used for *Agrobacterium* infiltration. For co-expression assays, bacterial suspensions carrying different constructs were mixed at a 1:1 ratio for infiltration.

### 2.4. Subcellular Localization Assays and Confocal Microscope

The eGFP fluorescence was excited by 488-nm laser lines, and the emission was detected at 490–540 nm. The YFP fluorescence was excited by 514-nm laser lines, and the emission was detected at 520–560 nm. The mCherry fluorescence was excited by 561-nm laser lines, and the emission was detected at 560–620 nm. The chlorophyll was excited by 488 nm laser lines, and the emission was detected at 640–720 nm. The images were taken by a laser scanning confocal microscope FV3000 (Olympus, Tokyo, Japan) and analyzed with FV31S-DT (Olympus).

### 2.5. Chloroplast Isolation

Intact chloroplasts were isolated from five-leaf *N. benthamiana* plants, as described previously [16]. In brief, leaves were homogenized in chloroplast isolation buffer (50-mM HEPES-KOH, pH 8.0, 5-mM MgCl_2_, 5-mM EDTA, pH 8.0, 5-mM EGTA, pH 8.0, 10-mM NaHCO_3_, and 0.33-M D-sorbitol) supplemented with the MedChemExpress Protease Inhibitor Cocktail (EDTA-free, mini-Tablet, 1 tablet per 10 mL) (MCE, Monmouth Junction, NJ, USA). The homogenate was then filtered through four layers of Miracloth and centrifuged at 800× *g* for 5 min at 4 °C. The pellets were suspended in isolation buffer and loaded onto a preset three-step Percoll gradient (10%:30%:80%) to separate intact and broken chloroplasts. Intact chloroplasts were taken from the green bands between the 30% and 80% layers after centrifugation at 2600× *g* for 30 min at 4 °C, and the obtained chloroplasts were washed twice with HS buffer (50-mM HEPES-KOH, pH 8.0, and 0.33-M D-sorbitol). Then, the purified chloroplasts were subjected to the Western blotting analysis.

### 2.6. Protein Import Assay

Prokaryotically expressed NSvc4 and eGFP controls were incubated with purified *N. benthamiana* chloroplasts for 30 min, followed by thermolysin treatment for 10 min on ice. Then, the chloroplasts were repurified by centrifugation at 2000× *g* for 5 min at 4 °C, and subsequently, the supernatant fraction and the pellet fraction containing the chloroplasts were subjected to Western blotting analysis.

### 2.7. Western Blotting and Antibodies

Plant total protein was extracted by using protein extraction buffer, as described previously [22]; then, equal amounts of protein were separated on 12.5% SDS-PAGE gels and blotted onto NC membranes. The membranes were probed with specific primary antibodies against eGFP (cat: AE012, ABclonal, Wuhan, China), FLAG tag (cat: F1804, Sigma, St. Louis, MO, USA), Myc tag (cat: A00704, GenScript, Piscataway, NJ, USA), RbcL (cat: ER1919-16, HUABIO, Hangzhou, China), and actin (cat: AC009, ABclonal, Wuhan, China). Primary antibodies against RSV NSvc4, RSV SP, PVX CP, and RSV CP were generated in our lab.

### 2.8. ROS Burst Measurements

Measurement of PTI-triggered ROS was performed as previously described [16]. Leaf discs from five-leaf *N. benthamiana* plants expressing different constructs were placed in white 96-well plates with water overnight. The next day, a solution containing 1-μM flg22, 100-mM luminol, and 20-mg/mL HRP was added to the leaf discs, and luminescence was immediately measured in a microplate reader for 30 min. For data analysis, both relative luminescence units (RLU) generated every minute upon flg22 treatment and total RLU over the duration of 30 min were calculated.

### 2.9. RNA Extraction and Real-Time Quantitative PCR

Total RNA was extracted from *N. benthamiana* plants using TRIzol reagent (Invitrogen, Carlsbad, CA, USA), as described previously [22]. Primer pairs specific to target the *NbPR1*, *NbPR2*, *NbWRKY12*, *NbGAPDH-A*, *NbPsbQ1*, and *RSV CP* genes were designed by using Oligo 7 (https://www.oligo.net/ accessed on 12 October 2020). RT-qPCR was performed using LightCycler 480 (Roche, Rotkreuz, Switzerland) as described previously [22].

### 2.10. Visulization of Chloroplast-Derived ROS

H_2_DCF-DA (2′7′-dichlorodihydrofluorescein diacetate) was used as a probe to detect photosynthesis-derived ROS in *N. benthamiana* leaves, as described previously [23]. Leaves expressing mCherry, mNSvc4-mCherry, and NSvc4-mCherry were treated with 5-mM H_2_O_2_ for 24 h, and then, 10-μM H_2_DCF-DA (Sigma-Aldrich, St. Louis, MO, USA, cat. no. D6883) was injected into leaves by using a needleless syringe. Five to ten hours later, the DCF signal were detected by confocal microscope under laser excitation at 488 nm and emission at 512–527 nm.

### 2.11. Co-IP Assay

Combinations of NSvc4-FLAG and NbGAPDH-A-Myc, NSvc4-FLAG, and GFP-Myc were co-expressed in five-leaf *N. benthamiana* plants. Total protein was extracted with the IP buffer, containing 40-mM Tris-HCl, pH 7.5, 150-mM NaCl, 5-mM MgCl_2_, 2-mM EDTA, 5-mM DTT, 1% Triton X-100, 5% glycerol, and MedChemExpress Protease Inhibitor Cocktail (EDTA free, mini-Tablet, 1 tablet per 10 mL), and the protein mixture was kept at 4 °C for 30 min. Protein samples were collected by centrifugation at 12,000× *g*, 4 °C for 15 min twice, and then immunoprecipitated with Anti-FLAG M2 Magnetic Beads (Sigma-Aldrich, St. Louis, MO, USA). The following procedures were then performed as previously described [21].

### 2.12. Mass Spectrometry

NSvc4-FLAG and CFP-FALG control were expressed in *N. benthamiana* plants, respectively. Protein samples were collected by immunoprecipitation, as described in Section 2.11. Then, the samples were subjected to a mass spectrometry analysis, which was carried out at AIMSMASS Co., Ltd. (Shanghai, China).

### 2.13. Prediction of Chloroplast Transit Peptide

The chloroplast transit peptide (cTP) was predicted by ChloroP (http://www.cbs.dtu.dk/services/ChloroP/ accessed on 4 July 2019).

### 2.14. Gene Sequences and IDs

The nucleotide sequences and IDs of the genes mentioned in this study are listed in Table S2, and the online genome database Sol Genomics Network (https://solgenomics.net/ accessed on 14 August 2020) for *N. benthamiana*.

### 2.15. Quantification of Fluorescence Intensity

The original confocal images acquired by Olympus FV3000 were sent to cellSens software (Olympus LS) for further image analysis. The images were not processed before analysis. The measurement and region of interest (ROI) tool was used to obtain the GFP fluorescence intensity in the chloroplast. The chloroplasts were circled as ROIs for measuring. The gray intensity value (mean) displayed in the measurement result represented the GFP fluorescence intensity. Three images were taken for each sample, and six chloroplasts in each image were circled as ROIs for measuring. The data were analyzed with GraphPad prism 8.

### 2.16. Isolation of Protoplast

For protoplast preparation, leaves infiltrated with NSvc4-eGFP were cut into small pieces and incubated with enzyme solution, as previously described [21]. After 2-h incubation, protoplasts were examined under a confocal microscope.

### 2.17. FM4-64 Treatment

FM4-64 (Invitrogen, cat. no. F34653) was diluted with DMSO to 5 mM for storage and then diluted with ddH_2_O to the working concentration 5 μM. *N. benthamiana* leaves were infiltrated with 5-μM FM4–64, and the leaves were kept at room temperature for 20–30 min to stain the membrane. The FM4–64 fluorescence was excited using 561 laser lines, and the emission was detected at 560–620 nm.

## 3. Results

### 3.1. N-Terminal 20 AAs Is Indispensable for NSvc4 to Localize to Chloroplasts

**Figure 1:**
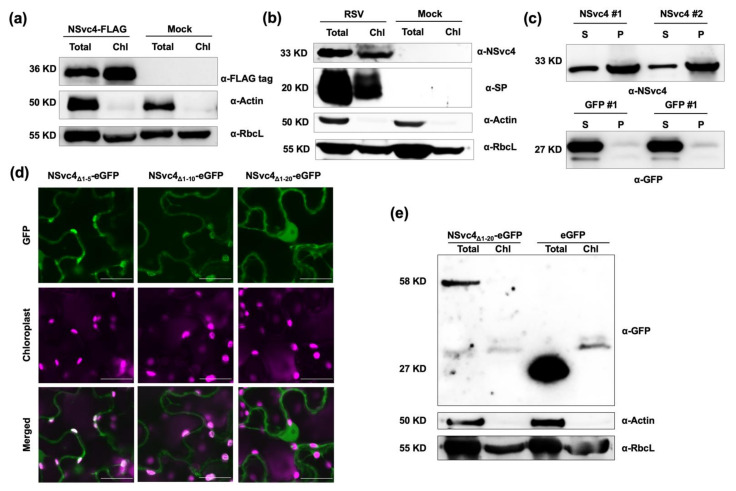
N-terminal 20 AAs are indispensable for NSvc4 to localize to chloroplasts. (**a**) Western blotting of total and chloroplast protein of *N. benthamiana* leaves transiently expressing NSvc4-FLAG and wild-type plants. Total, total protein. Chl, protein from the isolated chloroplasts fraction. Actin (cytoplasm marker). RbcL, RuBisco large subunit (chloroplast marker). (**b**) Western blotting of total and chloroplast protein from wild-type and RSV-infected *N. benthamiana* leaves. SP, disease-specific protein of RSV (RSV marker in the chloroplast). (**c**) *N. benthamiana* chloroplasts incubated with NSvc4 and eGFP were subjected to Western blotting. S, supernatant. P, pellet (intact chloroplast fraction). (**d**) Confocal images of *N. benthamiana* leaves infiltrated with agrobacteria carrying the constructs of NSvc4_Δ1–5_-eGFP, NSvc4_Δ1–10_-eGFP, and NSvc4_Δ1–20_-eGFP. Bars, 20 μm. (**e**) Western blotting of total and chloroplast protein of *N. benthamiana* transiently expressing NSvc4_Δ1–20_-eGFP and eGFP.

Previous studies have shown that NSvc4 is a chloroplast-targeting protein [19,20]. In order to further verify the accumulation of NSvc4 in the chloroplasts, chloroplast isolation experiments were conducted. Intact chloroplasts were purified from *N. benthamiana* leaves transiently expressing NSvc4-FLAG, and the presence of NSvc4 in chloroplasts was detected by Western blotting analysis (Figure 1a). We also found the presence of NSvc4 in the chloroplasts of RSV-infected *N. benthamiana* plants by using the same methods (Figure 1b). The above results show that NSvc4 is a chloroplast-targeting protein during RSV infection of *N. benthamiana*.

To test whether NSvc4 is capable of entering chloroplasts in vitro, the protein import assay was performed. As is shown in Figure 1c, after thermolysin treatment, NSvc4 was detected in the chloroplasts, although there was a weaker band in the supernatant fraction than in the chloroplast fraction, which was probably due to the incomplete import, as well as incomplete digestion. Whereas, for eGFP control, eGFP only existed in the supernatant but not in chloroplasts, which indicated that NSvc4 can enter the intact chloroplasts in vitro.

To further identify a non-chloroplast-localized NSvc4 mutant, we constructed several deletion mutants of NSvc4, including NSvc4_∆1–5_-eGFP, NSvc4_∆1–10_-eGFP, and NSvc4_∆1–20_-eGFP, which deleted N-terminal 1–5 AAs, 1–10 AAs, and 1–20 AAs, respectively. The subcellular localizations of these NSvc4 mutants were investigated. The accumulation of NSvc4_∆1–5_-eGFP and NSvc4_∆1–10_-eGFP in chloroplasts was observed; however, the chloroplast localization disappeared in the NSvc4_∆1–20_-eGFP mutant (Figure 1d). We also analyzed the GFP signal of the three truncated mutants in the chloroplasts. We found that the GFP signal of NSvc4_∆1–10_-eGFP in a chloroplast was lower than NSvc4_∆1–5_-eGFP but higher than NSvc4_∆1–20_-eGFP, which means the deletion of 5–10 AAs could also affect the chloroplast localization of NSvc4 to some degree (Figure S4). However, the mutant NSvc4_∆1–20_ almost abolished all the chloroplast localization; therefore, we regard NSvc4_∆1–20_ as a non-chloroplast-localized mutant of NSvc4, which is named mNSvc4. In addition, the chloroplast isolation assay also confirmed that NSvc4_∆1–20_-eGFP was almost undetectable in purified chloroplasts (Figure 1e).

### 3.2. The Localization of NSvc4 in Chloroplasts Inhibits Host Defense Response

**Figure 2:**
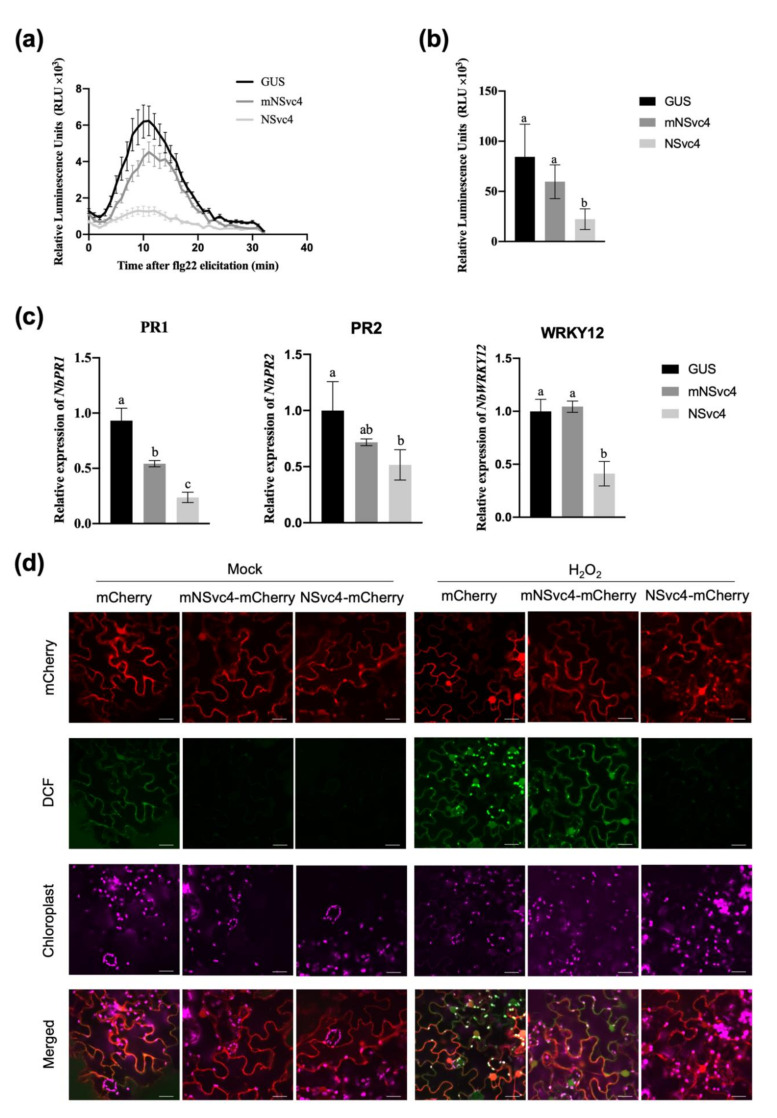
The localization of NSvc4 in chloroplasts inhibits host defense response. (**a**,**b**) ROS production as relative luminescence units (RLU) during 30 min in response to the elicitor flg22 in *N. benthamiana* leaves expressing GUS, mNSvc4, and NSvc4. Values represent the average of eight plants. Error bars represent SE. Different labels represent statistical significance (*p* < 0.01) by one-way ANOVA and Tukey’s test. (**c**) The relative expression levels of *NbPR1*, *NbPR2*, and *NbWRKY12* in *N. benthamiana* leaves transiently expressing GUS, mNSvc4, and NSvc4 after infiltration with flg22 were assayed by qRT-PCR, with the *NbActin* as a reference gene for normalization. The relative expression levels were normalized by setting the value of GUS control as 1.0 in each leaf (*n* = 3 biological replicates). Different labels represent statistical significance (*p* < 0.01) by one-way ANOVA and Tukey’s test. (**d**) *N. benthamiana* leaves infiltrated with agrobacterium expressing mCherry control, mNSvc4-mCherry, and NSvc4-mCherry were treated with ddH_2_O or 5-mM H_2_O_2_ for 24 h, followed by the treatment of H_2_DCF-DA. DCF signals were observed under confocal microscope. Bar, 20 μm.

Increasing studies have been reported that pathogens encode a variety of chloroplast-targeting effectors to inhibit the host defense. Therefore, we suspect that NSvc4 might play a similar role in regulating host immunity by locating in chloroplasts. To test our hypothesis, *N. benthamiana* leaves transiently expressing GUS, mNSvc4, and NSvc4 were treated with flg22, followed by ROS burst measurement. Compared with GUS control and mNSvc4, leaves expressing wild-type NSvc4 showed significantly a reduced ROS burst; however, leaves expressing mNSvc4 indicated slightly reduced ROS burst compared with the GUS control (Figure 2a,b). Meanwhile, the transcriptional levels of the defense-related genes were analyzed by RT-qPCR followed by infiltration of flg22. The expression levels of defense-related genes *NbPR1*, *NbPR2*, and *NbWRKY12* were much lower in plants expressing NSvc4 than in plants expressing GUS and mNSvc4 (Figure 2c). These results imply that NSvc4 could inhibit the host defense, which is likely dependent on the chloroplast localization of NSvc4.

Considering that chloroplast is an important factory of ROS, we want to know whether NSvc4 can inhibit the chloroplast-mediated defense by suppressing the ROS production and signal transduction in chloroplasts. We applied exogenous H_2_O_2_ to induce chloroplast-derived ROS production [24]. Leaves expressing mCherry, mNSvc4-mCherry, and NSvc4-mCherry were treated with double-distilled H_2_O (ddH_2_O) or 5-mM H_2_O_2_, and the accumulation of ROS derived from chloroplasts was detected with probe 2′7′-dichlorodihydrofluorescein diacetate (H_2_DCF-DA) under the confocal microscope. When we treated with ddH_2_O (mock), almost no signal of dichlorofluorescein oxide (DCF) was detected in leaves expressing mNSvc4-mCherry or NSvc4-mCherry, while only weak fluorescence in PM was observed in leaves infiltrated with mCherry control (Figure 2d). After being treated with 5-mM H_2_O_2_ for 24 h, strong fluorescence was observed mainly in the chloroplasts and PM in the leaves expressing mCherry and mNSvc4-mCherry, suggesting that chloroplast-derived ROS could be induced by exogenous H_2_O_2_. By contrast, only weak signals were detected in chloroplasts and PM of leaves infiltrated with NSvc4-mCherry (Figure 2d). The above results indicate that the accumulation of NSvc4 in chloroplasts could impair the chloroplast-mediated defense by inhibiting the production of photosynthetic ROS.

### 3.3. PVX-Mediated Expression of NSvc4 Promotes PVX Infection in N. benthamiana

**Figure 3:**
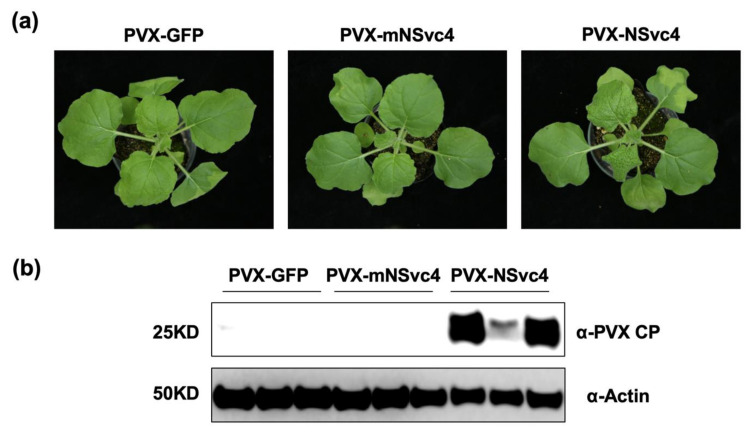
PVX-mediated expression of NSvc4 promotes PVX infection. (**a**) Symptoms elicited by PVX expressing GFP, mNSvc4, and NSvc4 at 5 dpi. (**b**) Western blotting of *N. benthamiana* leaves infected with PVX-GFP, PVX-mNSvc4, and PVX-NSvc4 to detect PVX accumulation by using anti-PVX CP antibodies. Actin was used as a loading control.

To investigate the roles of the chloroplast-localized NSvc4 in viral infection, we constructed PVX vectors to express the GFP control, mNSvc4, and wild-type NSvc4. By 5 days after agroinfiltration of *N. benthamiana* plants for the expression of GFP (PVX-GFP), mNSvc4 (PVX-mNSvc4), and NSvc4 (PVX-NSvc4), only plants infected with PVX-NSvc4 developed systemic symptoms in the upper emerging leaves (Figure 3a). The accumulation of PVX CP also indicated that only PVX-NSvc4 could cause systemic infection in the upper leaves at 5 dpi, but PVX-GFP and PVX-mNSvc4 failed to cause systemic infection at that time (Figure 3b). Therefore, these results indicate that the PVX vector expressing NSvc4 could promote viral infection, which may be at least partly due to the inhibition of the host defense by NSvc4.

### 3.4. Identification of Chloroplast-Related Host Factors Interacting with NSvc4

In order to determine how NSvc4 locates in chloroplasts and how it interferes with chloroplast-mediated defense, we performed the immunoprecipitation of NSvc4 followed by mass spectrometry (IP-MS) to identify host proteins interacting with NSvc4, especially chloroplast-related factors. The analysis of nano-liquid chromatography-tandem mass spectrometry (LC/MS/MS) showed that there might be 31 host proteins interacting with NSvc4 (Table S3), and among these, we focused on two chloroplast-related proteins, namely glyceraldehyde 3-phosphate dehydrogenase subunit A (NbGAPDH-A) and photosystem II subunit Q-1NbPsbQ1. A bioinformatics analysis revealed that both of the two proteins possess chloroplast transit peptides, and they also have been described as chloroplast-located proteins [25,26].

**Figure 4:**
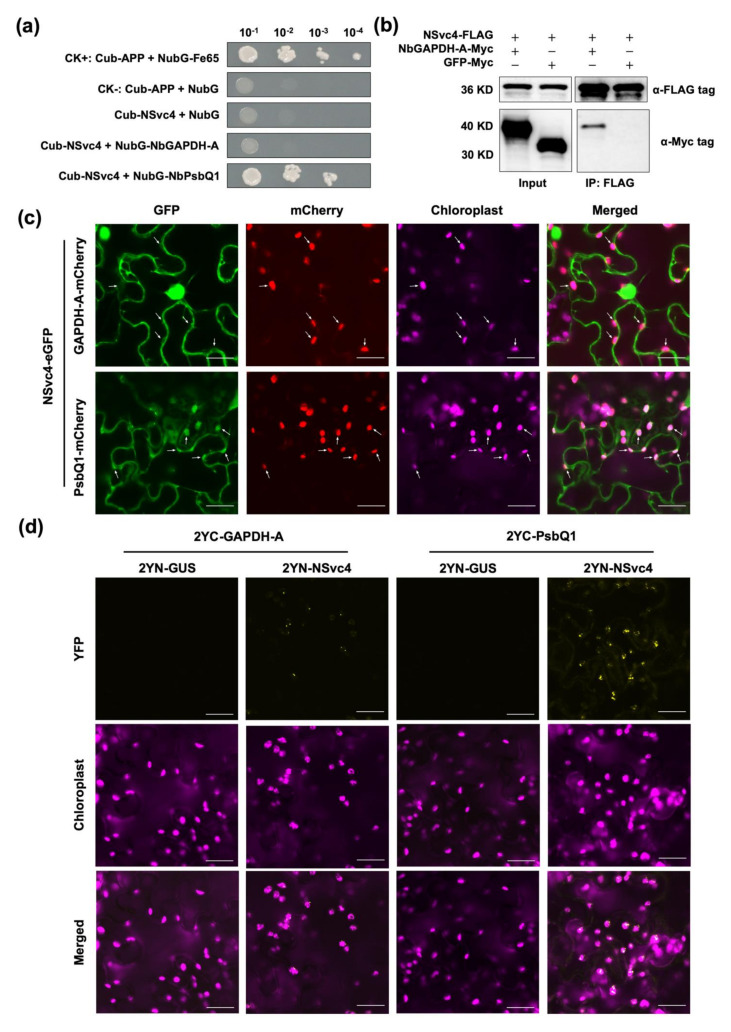
Identification of chloroplast-related host factors interacting with NSvc4. (**a**) Yeast split-ubiquitin assays. NSvc4 was fused to the C-terminal half (Cub), and NbGAPDH-A and NbPsbQ1 were, respectively, fused with the N-terminal half (NubG) of ubiquitin. Cub-APP co-transformed with NubG-Fe65 into yeast was used as a system positive control, Cub-APP co-transformed with NubG into yeast was used as a system negative control, and Cub-NSvc4 co-transformed with NubG into yeast was used as a negative control. A series of dilutions (10^−1^, 10^−2^, 10^−3^, and 10^−4^) are shown from left to right. (**b**) Co-IP assay of NSvc4 with NbGAPDH-A. NSvc4-FLAG was co-expressed with NbGAPDH-A-Myc or GFP-Myc in *N. benthamiana* leaves. Forty-eight hours later, the leaf protein extracts were incubated with FLAG magnetic beads. Samples before (Input) and after immunoprecipitation (IP) were analyzed by Western blotting using anti-FLAG or anti-Myc antibodies. (**c**) Confocal images of *N. benthamiana* leaves infiltrated with agrobacteria carrying the combination of NSvc4-eGFP with GAPDH-A-mCherry and PsbQ1-mCherry, respectively. Arrows indicate the chloroplast localization. Bars, 20 μm. (**d**) BiFC assays of NSvc4 with NbGAPDH-A and NbPsbQ1. Leaves were infiltrated with agrobacteria carrying the combinations of NSvc4 fused to the N-terminal part of YFP, with NbGAPDH-A and NbPsbQ1 fused to the C-terminal part of YFP, respectively, and GUS fused to the N-terminal part of YFP was used as a negative control. Samples were observed by laser confocal microscopy at 48 hpi. Bars, 20 μm.

To assess the interaction between NSvc4 and the two host proteins, the yeast split-ubiquitin assay was conducted. In these yeast split-ubiquitin assays, the C-terminal part of ubiquitin (Cub) was fused to NSvc4 to form bait, and the N-terminal part of ubiquitin (NubG) was fused with NbGAPDH-A and NbPsbQ1 to produce prey, respectively. Successful yeast growth was observed on selective media (SD/-Trp, -Leu, -His, and -Trp) when Cub-NSvc4 was co-expressed with NubG-NbPsbQ1, implying that NSvc4 is able to interact with NbPsbQ1 (Figure 4a). Noteworthily, the interaction between NSvc4 and NbGAPDH-A was not proven by the yeast spilt-ubiquitin assay; however, the interaction was confirmed by coimmunoprecipitation (Co-IP) assay, and we found that NbGAPDH-A-Myc could be coprecipitated with NSvc4-FLAG but GFP-Myc could not (Figure 4b).

To confirm the chloroplast localization of the NbGAPDH-A and NbPsbQ1, we transiently expressed NbGAPDH-A-mCherry and NbPsbQ1-mCherry in *N. benthamiana* leaves. Confocal microscope analysis showed that both of the two proteins are capable of localizing to chloroplasts (Figure S1). To address whether the two factors could affect the chloroplast localization of NSvc4, we co-expressed NbGAPDH-A-mCherry and NbPsbQ1-mCherry with NSvc4-eGFP in *N. benthamiana* leaves, respectively. We found that NbPsbQ1-mCherry colocalized with NSvc4-eGFP in the chloroplasts. Interestingly, when co-expressed with NbGAPDH-A-mCherry, the chloroplast localization of NSvc4-eGFP was significantly reduced (Figure 4c and Figure S5).

Bimolecular fluorescence complementation (BiFC) analysis was performed to verify in vivo interactions and study the subcellular localization of the interaction. NbGAPDH-A and NbPsbQ1 were cloned and fused to the C-terminal part of the YFP, respectively, while NSvc4 was fused to the N-terminal part of YFP. As is shown in Figure 4d, YFP fluorescence was observed in both combinations, which indicated that NSvc4 can interact with NbGAPDH-A and NbPsbQ1. The interaction between NbPsbQ1 and NSvc4 exhibited punctate spots, which were mainly in the chloroplast. The interaction of NbGAPDH-A and NSvc4 displayed punctate structures as well, which were adjacent to the chloroplasts, but future works need to be done to determine whether the interaction sites are in the chloroplasts or not.

### 3.5. Chloroplast-Related Factors Are Involved in Regulation of RSV Infection

**Figure 5:**
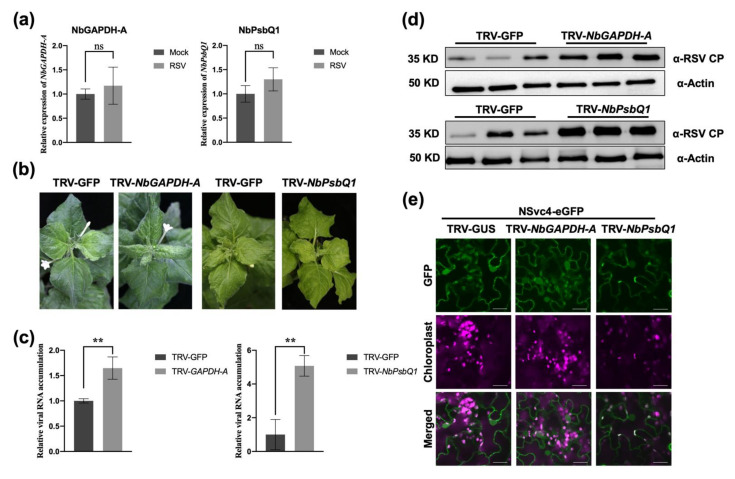
Chloroplast-related factors are involved in the regulation of RSV infection. (**a**) The relative expression levels of *NbGAPDH-A* and *NbPsbQ1* in RSV-infected or wild-type *N. benthamiana* leaves at 12 dpi were assayed by qRT-PCR, with *NbActin* as a reference gene for normalization. Data were analyzed by Student’s *t*-test, and asterisks denote significant differences between RSV-infected and mock plants (two-sided, *n* = 3; ns, not significant). (**b**) The symptoms of TRV-GFP, TRV-*NbGAPDH-A*, and TRV-*NbPsbQ1* RSV-inoculated plants at 11 dpi of RSV. (**c**) Detection of viral RNA accumulation in TRV-GFP, TRV-*NbGAPDH-A*, and TRV-*NbPsbQ1* RSV-inoculated plants. The leaves from RSV-infected plants in (**b**) were harvested. Total RNA was extracted for RT-qPCR to detect the transcription level of RSV CP. *NbActin* was used as a reference gene for normalization. Data were analyzed by Student’s *t*-test, and asterisks denote significant differences between TRV-GFP plants and TRV-*NbGAPDH-A* or TRV-*NbPsbQ1* plants (two-sided, *n* = 3, ** *p* < 0.01). (**d**) Detection of RSV CP accumulation in TRV-GFP, TRV-*NbGAPDH-A*, and TRV-*NbPsbQ1* RSV-inoculated plants. The leaves from RSV-infected plants in (**b**) were harvested. Total protein was extracted for Western blotting. The protein level of RSV CP represented the virus accumulation level. Actin was used as a loading control. (**e**) Subcellular localization of NSvc4-eGFP in *NbGAPDH-A*-silenced, *NbPsbQ1*-silenced, and TRV-GUS control *N. benthamiana* plants. Bars, 20 μm.

First, we studied the response of NbGAPDH-A and NbPsbQ1 upon RSV infection. RT-qPCR was performed to monitor the transcriptional changes of *NbGAPDH-A* and *NbPsbQ1* genes. The results showed that the relative expression levels of *NbGAPDH-A* and *NbPsbQ1* were not significantly different between wild-type plants and RSV-infected *N. benthamiana* plants (Figure 5a).

Next, we investigated whether the two factors play important roles in RSV infection. We silenced *NbGAPDH-A* and *NbPsbQ1*, respectively, by using tobacco rattle virus (TRV)-based VIGS. Compared with the control plants infected with TRV-GFP, the *NbGAPDH-A*-silenced plants displayed normal growth, but the *NbPsbQ1*-silenced plants showed slightly lower height (Figure S2). At 10 dpi, the silencing efficiency was detected, and RT-qPCR analysis showed that the two genes were both significantly downregulated in *N. benthamiana* plants (Figure S3).

Later, we inoculated *NbGAPDH-A*-silenced, *NbPsbQ1*-silenced, and TRV-GFP control plants with RSV, respectively. Following RSV inoculation, a noticeable vein yellowing symptom was observed in *NbGAPDH-A*-silenced and *NbPsbQ1*-silenced plants around 9–11 dpi, while TRV-GFP control plants had mild symptoms (Figure 5b), which displayed the obvious symptoms at 13 dpi. RT-qPCR and Western blotting analysis showed that the accumulation of the RSV CP in *NbGAPDH-A*-silenced and *NbPsbQ1*-silenced was significantly higher than that in TRV-GFP-infected plants at the transcriptional and translational levels (Figure 5c,d), which was related to the virus symptoms. These findings demonstrate that silencing of *NbGAPDH-A* and *NbPsbQ1* can both enhance RSV infection and symptom development, which means NbGAPDH-A and NbPsbQ1 could negatively regulate RSV infection.

To test whether NSvc4 is imported into the chloroplast by interacting with the two host factors, we transiently expressed NSvc4-eGFP in *N. benthamiana* plants silenced by *NbGAPDH-A* and *NbPsbQ1*. The subcellular localization analysis showed that the accumulation of NSvc4 in chloroplasts could still be observed in *NbGAPDH-A*-silenced and *NbPsbQ1*-silenced plants (Figure 5e), indicating that the ability of NSvc4 to be imported into the chloroplast is not affected by silencing of the two host factors.

## 4. Discussion

In recent years, an increasing number of studies have supported that chloroplast is not only the energy producer for plants but also plays a key role in plant defense against pathogen infection [27]. Chloroplast-dependent immunity may be interfered by effectors released by pathogens, such as C4 of TYLCV and Pst_12806 encoded by wheat stripe rust *Puccinia striiformis* f. sp. *tritici* [16,23]. It has been reported that RSV infection could lead to the alteration of normal chloroplast structure and function in *N. benthamiana*, and both SP and NSvc4 of RSV can localize to chloroplasts [18,20]. SP targets the PS II in the chloroplast and interacts with one of the OEC members, PsbP [18]. The interaction between SP and PsbP enhances the virus symptom induction [18]. NSvc4 is also a chloroplast-targeting protein, and the N-terminal 1–73 AAs are necessary and sufficient for chloroplast localization [20]. However, the function of NSvc4 localizing to the chloroplast is unclear. In this study, we revealed that expression of NSvc4 in *N. benthamiana* leaves inhibited the chloroplast-dependent defense. Compared with plants expressing GUS and non-chloroplast-located NSvc4 mutant, plants expressing wild-type NSvc4 showed impaired immune responses, including reduced ROS outbreak (Figure 2a), inhibition of defense-related gene expression (Figure 2c), and defects in chloroplast-derived defense signals (Figure 2d).

In our study, we showed that the PVX-mediated expression of NSvc4 can promote PVX infection in *N. benthamiana* (Figure 3a,b). Previous research found that PVX-NSvc4_106–286_, which was not predicted to localize to chloroplast, could also elicit similar symptoms to PVX-NSvc4 [20]. The different results might due to the different times that the symptoms were recorded, because we observed the PVX symptoms at 5 dpi, while the virus symptoms were recorded at 20 dpi in previous research. We found that the symptom elicited by PVX-NSvc4 was much serious than PVX-mNSvc4 at 5 dpi, while, at 20 dpi, the symptoms elicited by both became similar. Therefore, we speculate that the suppression of chloroplast-mediated defense caused by chloroplast-localized NSvc4 was at the earlier stage of virus infection. In addition, NSvc4 has multiple subcellular localizations in infected cells. Therefore, it is possible that NSvc4 has the function to suppress host immunity by locating at other organelles with other mechanisms.

Some pathogen effectors are transported into the chloroplast by using cleavable transit peptides, which can be recognized by the host transport pathway involving translocons of the outer and inner chloroplast membranes (TOC and TIC) [28]. However, the chloroplast transit peptide was predicted to be absent in NSvc4 by the bioinformatics analysis, and the N-terminal of NSvc4 would not be cleaved after being imported into the chloroplast (Figure 1a,b). Therefore, NSvc4 may be transported into the chloroplast via a noncanonical pathway. There are several alternative ways to assist the chloroplast targeting of host proteins. For instance, some outer envelope membrane (OEM) proteins, lacking cleavable transit peptides, are targeted at the chloroplast by encoding transmembrane domains, which can be recognized by OEM receptors [29]. Another example is the chloroplast inner membrane protein quinone oxidoreductase homologue (QORH), which possesses a non-cleavable targeting signal and localizes to the inner membrane without the interaction with the TOC complex [30,31]. Alternatively, it has been reported that viral proteins target chloroplasts by interacting with the host chloroplast-targeting proteins, and one such example is PVX CP. PVX CP does not have a transit peptide but enters the chloroplast by interacting with the host plastocyanin transit peptide [32]. In this study, the N-terminal 1–73 AAs of NSvc4 are sufficient for NSvc4 targeting to the chloroplast. Therefore, we speculate that there might be unknown host factors interacting with the N-terminal 1–73 AAs to transport NSvc4 into the chloroplast. The pathway that NSvc4 adopts to achieve chloroplast import is of significance to have further investigations come up with disease-resistance strategies in the future.

The interaction between host factors and viral proteins is indispensable in the life cycle of viruses [33]. Previous studies have demonstrated that RSV-encoded proteins can interact with multiple host factors to promote viral infection [17]. NSvc4 is the movement protein of RSV, and NSvc4 can interact with the plant remorin protein to promote virus movement by increasing the permeability of PD [21]. Moreover, NSvc4 can also interact with NbMIP1 in *N. benthamiana* and OsDjA5 in rice to prevent being degraded by unfolded protein response-induced autophagy [22]. Chloroplast-related host factors also play a variety of roles in virus infection [11]. Here, we determined that NbGAPDH-A, and NbPsbQ1 can interact with NSvc4.

GAPDHs are important enzymes involved in glycolysis and gluconeogenesis [34], and higher plants possess multiple GAPDH isoforms, including cytosolic glycolytic GAPDHs, chloroplastic photosynthetic GAPDHs, plastid glycolytic GAPDHs, and the NADP-dependent non-phosphorylated cytoplasmic GAPDH (NP-GAPDH). GAPDH-A and another subunit, GAPDH-B, are both located in the chloroplast, playing important roles in the Calvin–Benson cycle [25]. It has been proven that NbGAPDH-A can interact with the MP of red clover necrotic mosaic virus (RCNMV) in the cortical viral replication complex (VRC). NbGAPDH-A plays a positive role in the intercellular movement of RCNMV, and the silencing of *NbGAPDH-A* inhibits MP localization to the cortical VRC and reduces virus accumulation [35]. Our study shows that NSvc4 can interact with NbGAPDH-A in vivo (Figure 4b). Interestingly, the accumulation of NSvc4 in chloroplasts was dramatically diminished in NbGAPDH-A-overexpressing leaves (Figure 4c), suggesting the overexpression of NbGAPDH-A can inhibit the suppression of chloroplast-dependent immunity caused by NSvc4. Further evidence shows that silencing of *NbGAPDH-A* promotes RSV infection (Figure 5b), which is probably due to increased accumulation of NSvc4 in chloroplasts. Nevertheless, it is still unclear why the overexpression of NbGAPDH-A inhibits the chloroplast localization of NSvc4.

PsbQ is located in the thylakoid membrane, together with the PsbO, PsbP, and PsbR proteins, to form the extrinsic subunits of the oxygen-evolving complex (OEC) of Photosystem II (PS II) [26]. In higher plants, there are two genes encoding PsbQ, namely *PsbQ1* and *PsbQ2* [26], and previous studies have shown that PsbQ is crucial for the normal function of the PS II [36,37]. PsbQ1 is one of the key components of the OEC of PS II, and evidence showed that OEC plays a significant role in response to biotic or abiotic stresses [9,38]. HopN1 of *Pseudomonas syringae* pv. tomato DC3000 can target the OEC and interact with PsbQ, and the interaction disturbs the host PS II activity, leading to the reduced chloroplast-derived ROS burst, which is due to the proteolysis of PsbQ caused by HopN1 [39]. PsbQ plays a vital role in maintaining OEC stability, and OEC is one of the main places where ROS are produced in chloroplasts. The downregulation of *NbPsbQ1* facilitates RSV infection (Figure 5b); therefore, NSvc4 probably inhibited the production of chloroplastic ROS by disturbing the normal function of NbPsbQ1. In addition, previous research has demonstrated that the SP protein of RSV can interact with another member of OEC, PsbP. This protein–protein interaction affects the subcellular localization of PsbP and may indirectly alter the structure and function of the chloroplasts in infected cells to promote RSV infection. Since NSvc4 and SP can both target the OEC, the OEC may play a significant role in RSV infection. Furthermore, the interaction between viruses and the OEC has been found in several other cases. For example, the CP of alfalfa mosaic virus can interact with PsbP to facilitate virus infection [40]; the TGB3 protein of *Alternanthera* mosaic virus can interact with PsbO to increase virus accumulation and induce virus symptoms [41]. These findings imply that the OEC might play an essential role in chloroplast-mediated defense and may be the main target of pathogen-encoded proteins in chloroplast.

In our proposed model, Nsvc4 accumulates in the chloroplast by using an undefined strategy and, consequently, suppressing the chloroplast-mediated immunity to promote virus infection. In addition, in our research, two chloroplast-targeting host factors, NbGAPDH-A and NbPsbQ1, have been identified to interact with NSvc4. However, the molecular mechanisms about the interactions between NSvc4 and the chloroplast-related factors need to be further studied to find out how NSvc4 targets chloroplasts and how it interferes with the chloroplast-mediated defense.

## Data Availability

All the data used in this study are already provided in the manuscript in its required section. There is no underlying data available.

## Supplementary Materials

The following are available online at https://www.mdpi.com/article/10.3390/v14010036/s1: Figure S1: The chloroplast localization of NbGAPDH-A and NbPsbQ1. Figure S2: The phenotype of silencing NbGAPDH-A and NbPsbQ1 by TRV-based VIGS. Figure S3: Quantification of the gene silencing efficiency of NbGAPDH-A-silenced and NbPsbQ1-silenced plants. Figure S4: Quantification of the GFP fluorescence intensity in chloroplasts of NSvc4 truncated mutants. Figure S5: Quantification of GFP fluorescence intensity in chloroplasts of NSvc4-eGFP when co-expressed with GAPDH-A-mCherry and PsbQ1-mCherry. Figure S6: Confocal images showing NSvc4 can localize to PD, ER, and PM. Table S1: The list of primers used in this study. Table S2: IDs and sequences of the genes used in this study. Table S3: The list of host factors identified to interact with NSvc4 via IP-MS.
