# Supplementary material for: NSvc4 Encoded by Rice Stripe Virus Targets Host Chloroplasts to Suppress Chloroplast-Mediated Defense
Source: Viruses. 2021 Dec 24;14(1):36. doi: 10.3390/v14010036 (PMC8778898; doi:10.3390/v14010036)
Supplement: Supplementary file 1 [file viruses-14-00036-s001.zip › Supplementary material.pdf]

SUPPLEMENTARY FIGURE S1

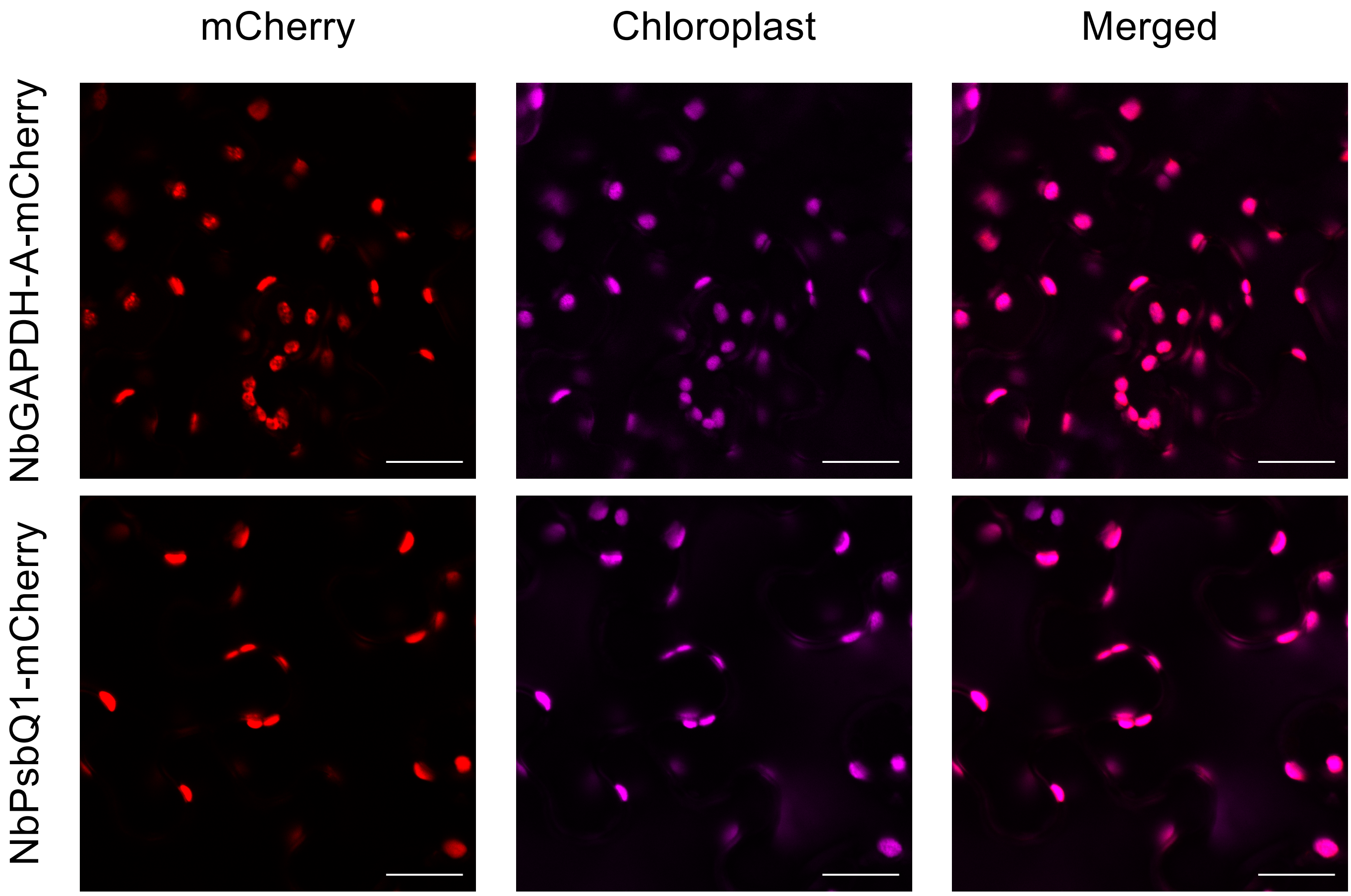

Figure S1. The chloroplast localization of NbGAPDH-A and NbPsbQ1. Confocal images of *N. benthamiana* leaves infiltrated with agrobacteria carrying NbGAPDH-A-mCherry and NbPsbQ1-mCherry, respectively. Bars, 20 μm.

SUPPLEMENTARY FIGURE S2

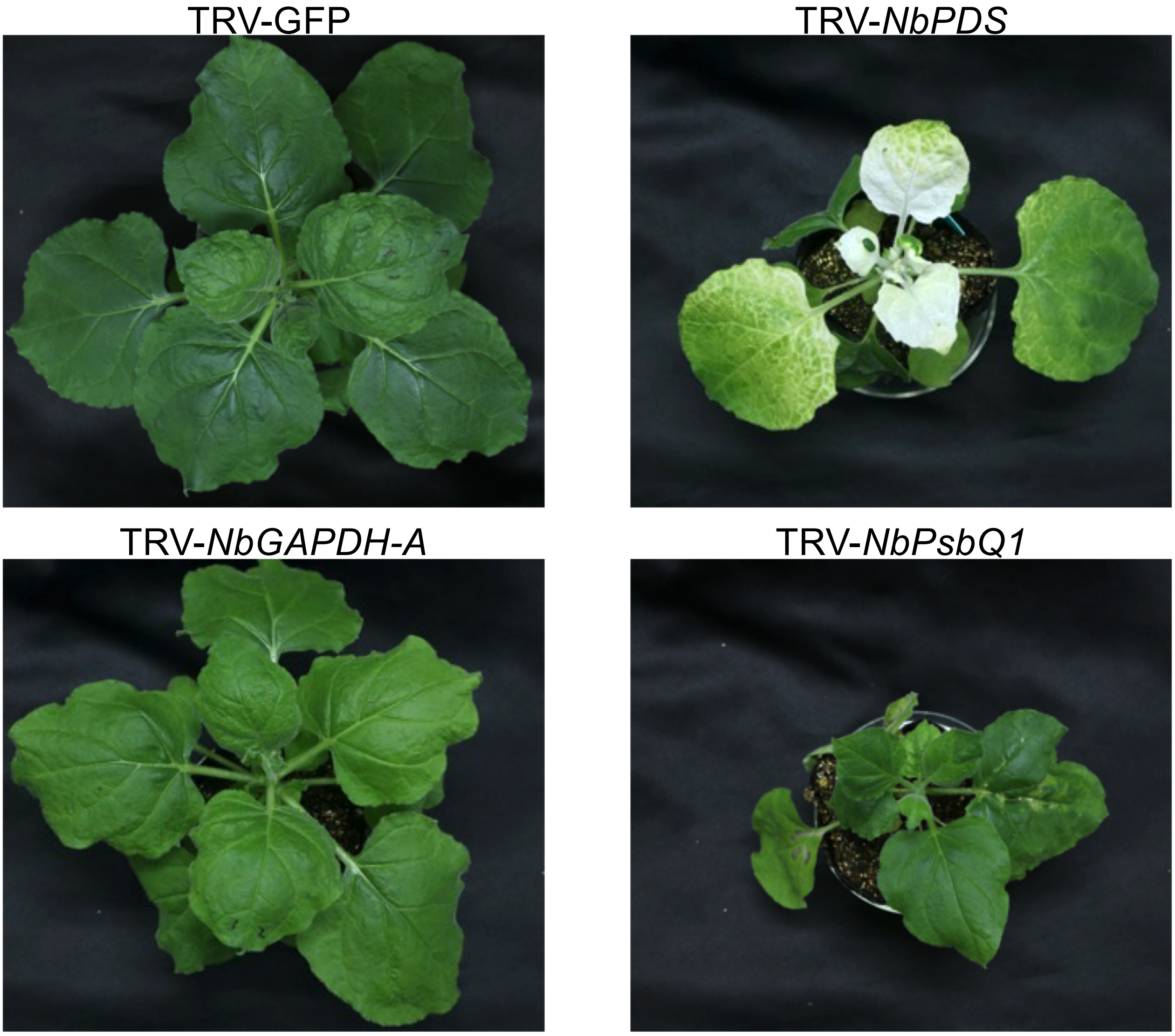

Figure S2. The phenotype of silencing *NbGAPDH-A*, *NbPsbQ1* by TRV-based VIGS. The image was taken at 10 days after inoculation of TRV.

SUPPLEMENTARY FIGURE S3

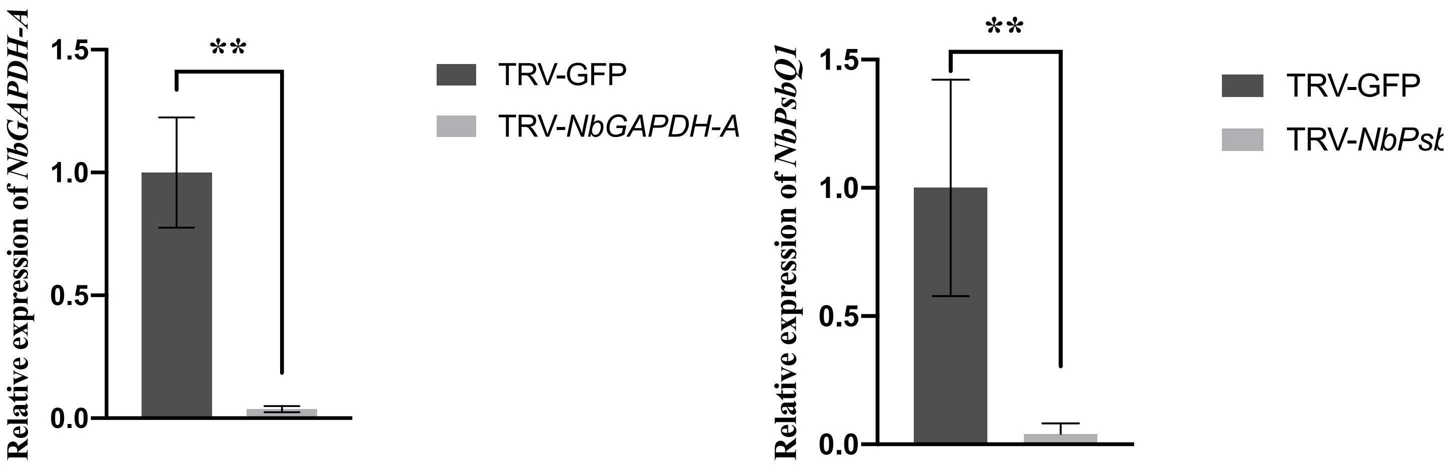

Figure S3. Quantification of gene silencing efficiency. RT-qPCR analysis of TRV-*NbGAPDH-A* and TRV-*NbPsbQ1* inoculated *N. benthamiana*. The leaves from TRV-*NbGAPDH-A* and TRV-*NbPsbQ1* inoculated plants were harvested for total RNA extraction. RT-qPCR analysis was performed to test the transcriptional level of *NbGAPDH-A*, and *NbPsbQ1*. *NbActin* was used as an internal reference in relative quantification. Student’s t-test was performed, and asterisks denote significant differences between TRV-GFP and TRV-*GAPDH-A*, or TRV-*PsbQ1* plants inoculated plants (n=3, two-sided, **P < 0.01, ****P < 0.0001).

SUPPLEMENTARY FIGURE S4

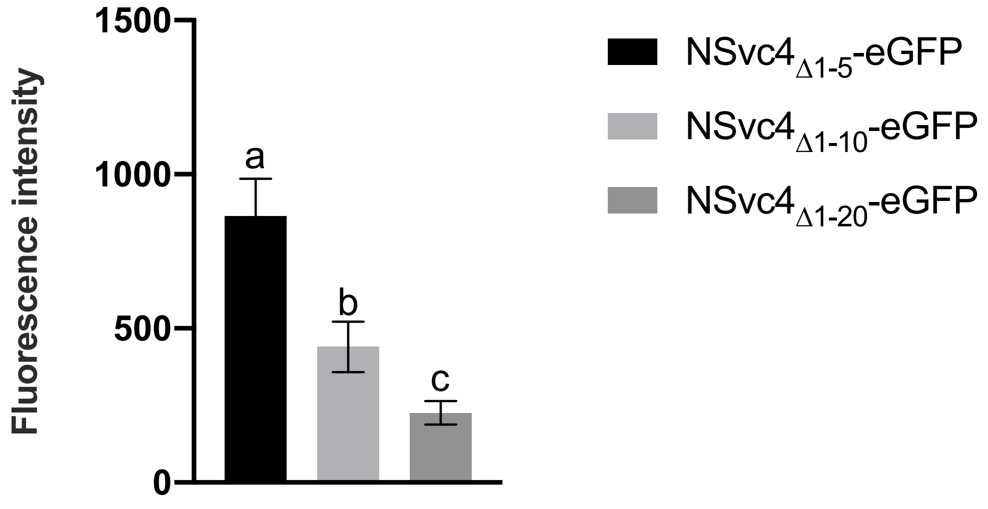

Figure S4: Quantification of GFP fluorescence intensity in chloroplasts of NSvc4$_{\Delta 1\text{-}5}$-eGFP, NSvc4$_{\Delta 1\text{-}10}$-eGFP, and NSvc4Δ1-20-eGFP. Different labels represent statistical significance ($p < 0.0001$), one-way ANOVA and Tukey's test (n = 6 biological replicates).

SUPPLEMENTARY FIGURE S5

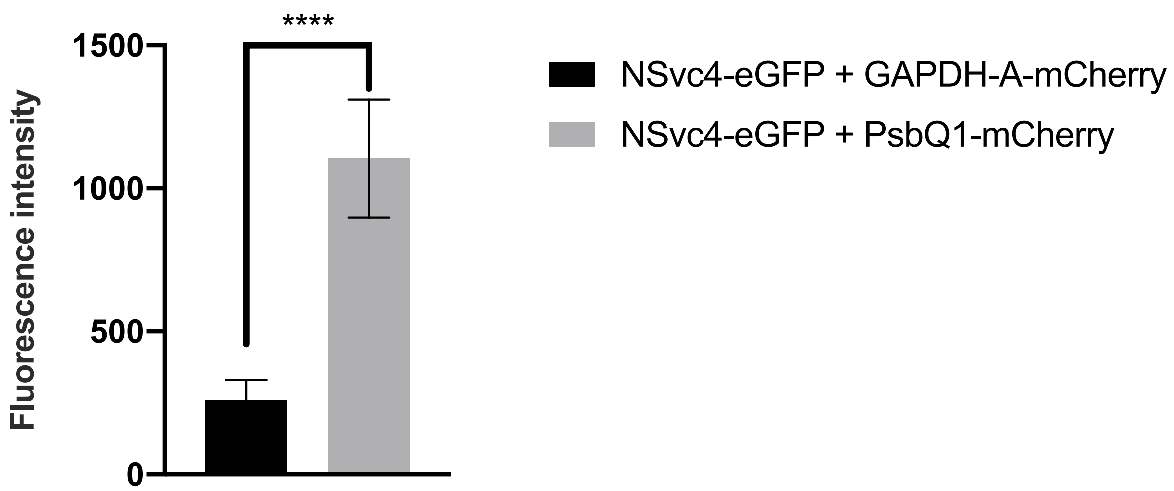

Figure S5: Quantification of GFP fluorescence intensity in chloroplasts of NSvc4-eGFP when co-expressed with GAPDH-A-mCherry and PsbQ1-mCherry. Data were analyzed by Student's t-test, and asterisks denote significant differences between the two situations (two-sided, n=6, ****$P < 0.0001$)

SUPPLEMENTARY FIGURE S6

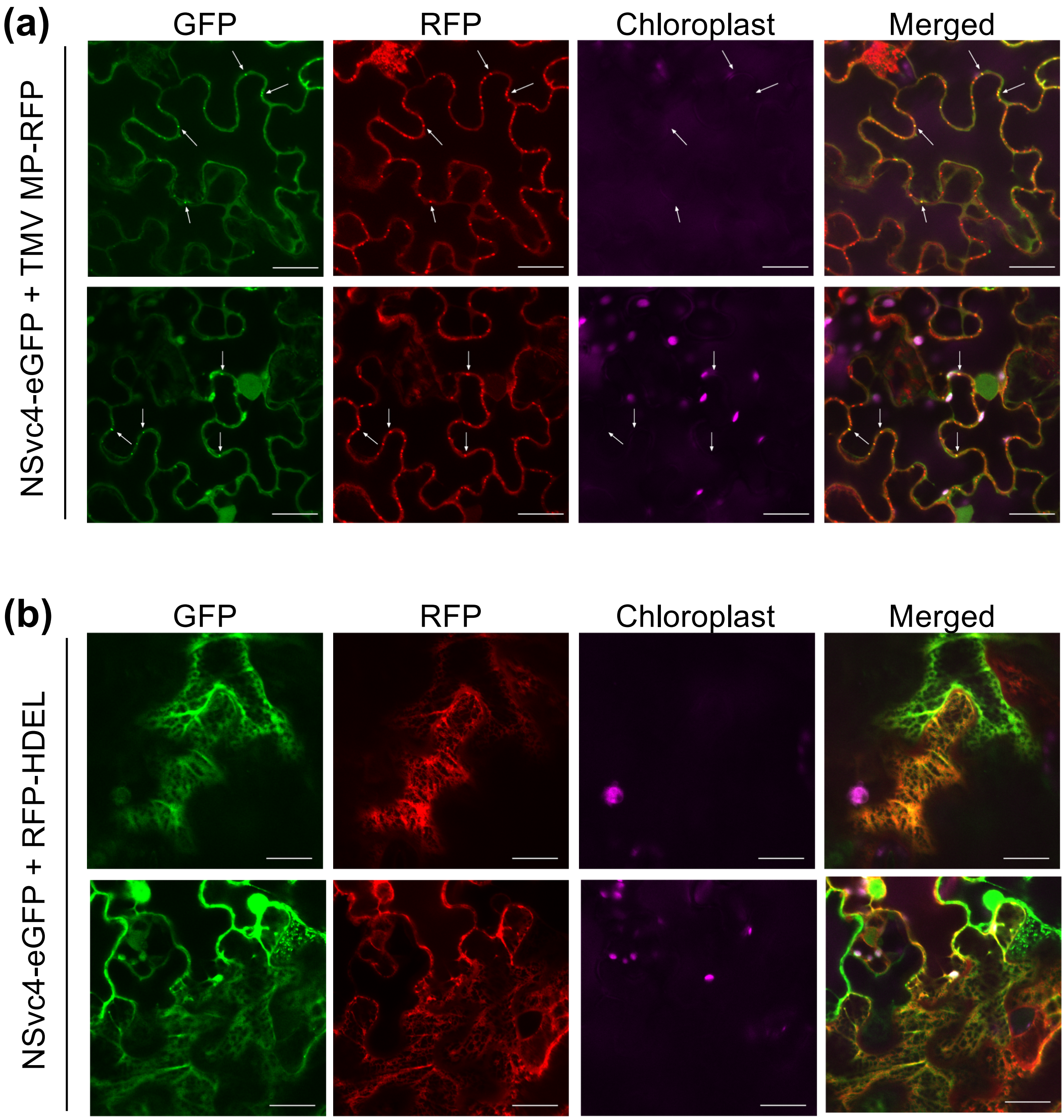

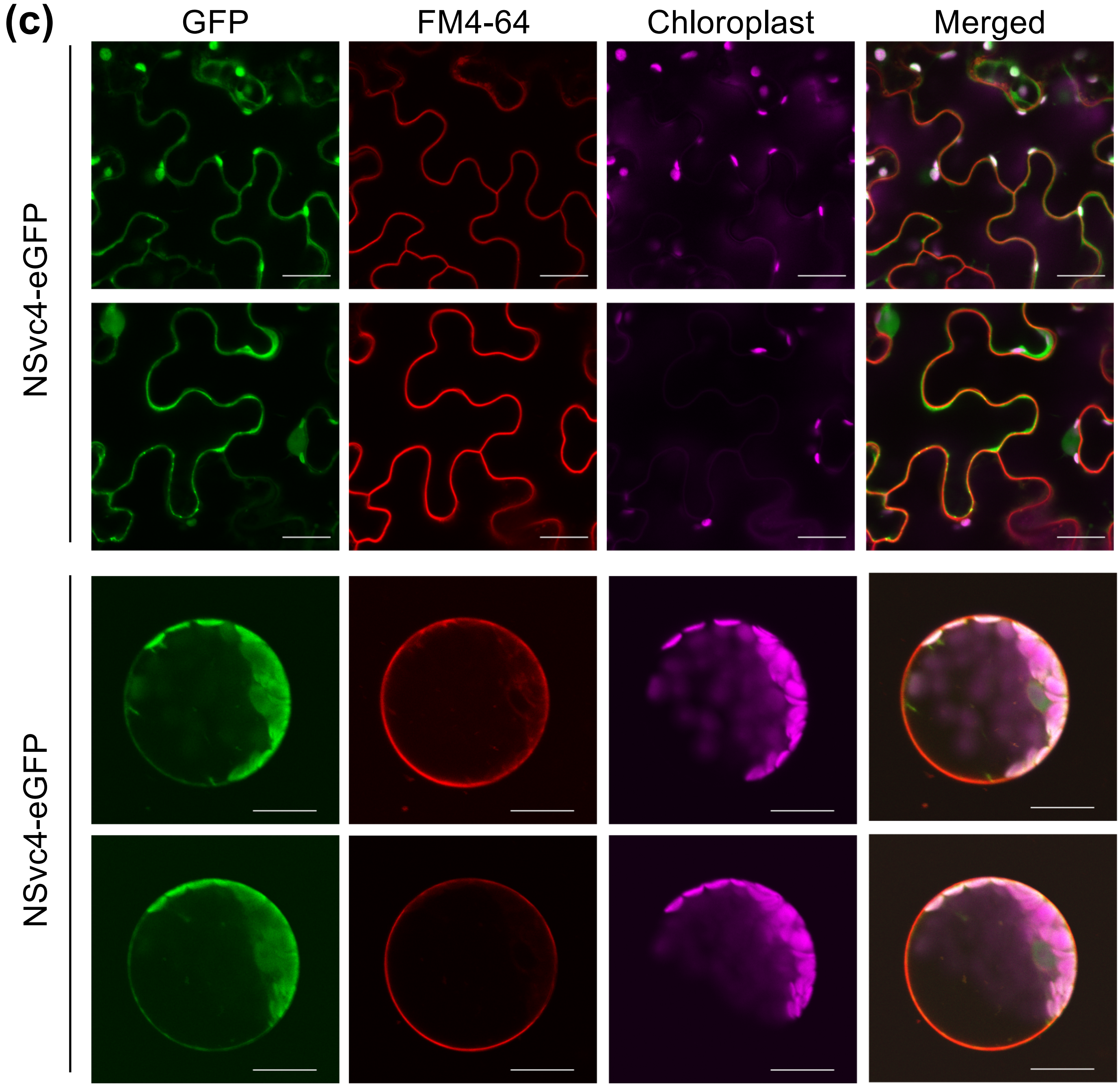

Figure S6. Confocal images showing NSvc4 can localize to PD, ER, and PM**. (a)**The co-localization of NSvc4-eGFP and TMV MP-RFP (PD marker). Confocal images of *N. benthamiana* leaves infiltrated with agrobacteria carrying NSvc4-eGFP and TMV MP-RFP. Bars, 20 μm. **(b)** The co-localization of NSvc4-eGFP and RFP-HDEL (ER marker). Confocal images of *N. benthamiana* leaves infiltrated with agrobacteria carrying NSvc4-eGFP and RFP-HDEL. Bars, 20 μm. **(c)** The co-localization of NSvc4-eGFP with FM4-64 fluorescence. Confocal images of *N. benthamiana* expressing NSvc4-eGFP stained with FM4-64 (dye to stain PM). The upper two rows were images taken from leaves, the lower two rows were images taken from protoplasts. Bars, 20 μm.

SUPPLEMENTARY TABLE S1: The list of primers used in this study

Name	Sequence (5'-3')	Application
pCV-NSvc4-F	ACTCTAGACCCCTGGGATCCATGGCTTTGTCTCGACTTTT	
pCV-NSvc4-R	CGATCGGGGAATTCGAGCTCTTACATGATGACAGAAACTT	
pCV-mNSvc4-F	CTAGACCCCTGGGATCCatgGAGGAGTCCCAAAAGAGAGT	
pCV-mNSvc4-R	CGATCGGGGAATTCGAGCTCTTACATGATGACAGAAACTT	
pGD-NSvc4-eGFP-F	ctacaaatctatctctaacaATGGCTTTGTCTCGACTT	
pGD-NSvc4-eGFP-R	tcctcgcccttgctcaccatCATGATGACAGAAACTTC	
pGD-NSvc4 (1-73)-eGFP-F	ctacaaatctatctctaacaATGGCTTTGTCTCGACTT	
pGD-NSvc4 (1-73)-eGFP-R	tcctcgcccttgctcaccatCATATCATACTTGTTAAC	
pGD-NSvc4 (1-73Δ)-eGFP-F	ACGAGCTCGGTACCCGGatgTTCATAGCCAAACAGGACTA	
pGD-NSvc4 (1-73Δ)-eGFP-R	ctcaccatGTCGACTCTAGACATGATGACAGAAACTTCAG	
pGD-NSvc4 (1-5Δ)-eGFP-F	GGGACGAGCTCGGTACCCGGatgCTTTTGTCCACTTCAAA	
pGD-NSvc4 (1-5Δ)-eGFP-R	ctcaccatGTCGACTCTAGACATGATGACAGAAACTTCAG	
pGD-NSvc4 (1-10Δ)-eGFP-F	GGGACGAGCTCGGTACCCGGatgAAAAGTAAGGTACTCTA	
pGD-NSvc4 (1-10Δ)-eGFP-R	ctcaccatGTCGACTCTAGACATGATGACAGAAACTTCAG	Plasmid construction
pGD-NSvc4 (1-20Δ)-eGFP-F	GGGACGAGCTCGGTACCCGGatgGAGGAGTCCCAAAAGAG	
pGD-NSvc4 (1-20Δ)-eGFP-R	ctcaccatGTCGACTCTAGACATGATGACAGAAACTTCAG	
pGD-NSvc4-mCherry-F	ctacaaatctatctctaacaATGGCTTTGTCTCGACTT	
pGD-NSvc4-mcherry-R	tcctcgcccttgctcaccatCATGATGACAGAAACTTC	
pGD-mNSvc4-mCherry-F	caaatctatctctaacaatgGAGGAGTCCCAAAAGAGAGT	
pGD-mNSvc4-mCherry-R	tcctcgcccttgctcaccatCATGATGACAGAAACTTC	
pgR106-mNSvc4-F	ctagcatcgattggcgcgccatgGAGGAGTCCCAAAAGAG	
pgR106-mNSvc4-R	aagcttatcggcggtcgacttaCATGATGACAGAAACTTCAG	
pPR3N-NbGAPDH-A-F	attacgctggatccgaattcatggcttcggctgctctc	
pPR3N-NbGAPDH-A-R	atcgatgctagcccatggtttccactggtttgcaacaatgtc	
pPR3N-NbPsbQ1-F	attacgctggatccgaattcatggctcatgctatggcttca	
pPR3N-NbPsbQ1-R	taatcgatgctagcccatggttaaccaagtttggccaaaacatcat	
pGD-NbGAPDH-A-Myc-F	ttacgaacgatagggatccATGGCTTCGGCTGCTCTCT	
pGD-NbGAPDH-A-Myc-R	agctctgtcgacatctagaTTTCCACTGGTTTGCAACAAT	
pGD-NbGAPDH-A-eGFP/mCherry-F	tctctacaaatctatctctATGGCTTCGGCTGCTCTCT	
pGD-NbGAPDH-A-eGFP/mCherry-R	ctcgcccttgctcaccatTTTCCACTGGTTTGCAACAAT	

pGD-NbPsbQ1-eGFP/mCherry-F	ctctacaaatctatctctatggctcatgctatggcttc	
pGD-NbPsbQ1-eGFP/mCherry-R	CTCGCCCTTGCTCACCATaccaagtttggccaaaacat	
2YC-NbGAPDH-A-F	ttaattaaATGGCTTCGGCTGCTCT	
2YC-NbGAPDH-A-R	GGCGCGCCTTTCCACTGGTTTGCAACAAT	
2YC-NbPsbQ1-F	TACGAACGATAGTTAATTAAatggctcatgctatggcttc	
2YC-NbPsbQ1-R	CCTCCACTAGTGGCGCGCCcaccaagtttggccaaaacat	
TRV2-NbGAPDH-A-F	ggttaccgaattctctagaTATTGGAGGAGGGAACAAC	
TRV2-NbGAPDH-A-R	gacatgcccgggcctcgagCAGGGGAGGTTGACAGG	
TRV2-NbPsbQ1-F	aggttaccgaattctctagactcagcattagagcccaaca	
TRV2-NbPsbQ1-R	acatgcccgggcctcgagattcttgacgctaacaatctcc	
qPCR-NbActin-F	GTTGCTATACAAGCTGTTCTCTCG	
qPCR-NbActin-R	GTCAAGACGAAGAATGACATGTGG	
qPCR-NbPR1-F	CGACCAGGTAGCAGCCTATG	qPCR
qPCR-NbPR1-R	TCTCAACAGCCTTAGCAGCC	
qPCR-NbPR2-F	GGGCTGTTAATTTGCAGTATCC	
qPCR-NbPR2-R	GGTTTATAACATCTTGGTCTGATGG	
qPCR-NbWRKY12-F	CTCATCAGCTAGTTCATTTGATGC	
qPCR-NbWRKY12-R	AGCTCGGTCTTTGTTCTAAAAGC	
qPCR-NbGAPDH-A-F	GTGTGCCAACCCCTAACGTCT	
qPCR-NbGAPDH-A-R	ACCTTAACCATGTCATCTCCCAT	
qPCR-NbPsbQ1-F	agaataccttcgctatgacct	
qPCR-NbPsbQ1-R	tgttcttggtctttgctgcat	

SUPPLEMENTARY TABLE S2: IDs and sequences of genes used in this study

- **IDs and sequences of genes used in this study**

RSV NSvc4
ATGGCTTTGTCTCGACTTTTGTCCACTTCAAAAAGTAAGGTACTCTATGATGACCTTAGTGAGGA
GTCCCAAAAGAGAGTTGATAATAAGAATAGGAAATCTCTAGCTCTCTCCAAGAGGCCTCTGAAC
CAGGGTAGGGTTACGATTGACCAAGCTGCCACAATGCTTGGATTGGAACCTTTCAGCTTCTCAG
ATGTCAAGGTTAACAAGTATGATATGTTCATAGCCAAACAGGACTATTCTGTGAAGGCCCATAG
GAAAGCAACTTTCAACATACTTGTTGATCCATATTGGTTTCATCAACCTCTCACCCATTACCCATT
CTTTAGAGTGGCAACTTTTGCTATGGTCTGGATTGGAATTAAGGGTAGAGCTAGTGGAATAACTA
CCCTCCGCATAATAGACAAGTCCTATGTGAACTCCTCAGATCAAGTTGAGGTAGAAGTTCGCTA
CCCAATTAGCAAGAATTTTGCTGTCTTGGGTTCTCTAGCGAATTTTCTGGCTTTGGAGGATAAGC
ATAATCTTCAAGTGTCCGTATCTGTTGATGACAGCTCAGTGCAGAACTGTGTCATCTCTAGAACT
CTTTGGTTCTGGGGGATTGAGAGGACTGATCTGCCAGTGTCCATGAAGACTAATGACACTGTGA
TGTTTGAATTTGAACCACTAGAAGATAAAGCTATAAACCATTTGTCCAGCTTCAGTAATTTTACG
ACTAATGTTGTTCAGAAAGCTGTTGGAGGTGCTTTCACTAGCAAGAGCTTCCCTGAGCTAGACA
CTGAGAAGGAGTTCGGTGTGGTGAAGCAGCCAAAGAAGATACCCATCACAAAGAAATCAAAAT
CTGAAGTTTCTGTCATCATGTAG

NbGAPDH-A (Niben101Scf05188g03030.1)
ATGGCTTCGGCTGCTCTCTCAGTAGCCAACTCTTCTCTTCAGGTCAGCAACAAAGGATTCTCTGA
ATTCTCAGGGCTGCGCACGTCATCAGCTATTCCATTCGGAAGGAAAACCAACGATGACTTGCTC
TCTGTTGTTGCCTTCCAAACCTCTGTTATTGGAGGAGGGAACAACAAGAGGGGAGTAGTGGAG
GCCAAGTTGAAAGTGGCCATCAATGGATTTGGAAGAATTGGAAGGAATTTCTTGAGGTGTTGGC
ATGGTAGGAAAGACTCTCCCCTTGATGTCATTGCCATCAATGACACTGGTGGTGTCAAGCAAGC
CTCTCACCTTCTCAAATATGACTCCACCCTTGGCATCTTTGATGCTGATGTCAAGCCCGTCGGCA
CTGACGGCATCTCCGTCGACGGAAAAGTCATCCAAGTCGTCTCCGACCGCAACCCTGTCAACCT
CCCCTGGGGAGATCTTGGGATTGACTTGGTGATAGAAGGTACCGGAGTGTTTGTCGACAGAGA
AGGTGCCGGAAAACACATCCAGGCCGGAGCCAAGAAGGTGCTCATCACCGCTCCAGGAAAAG
GTGACATCCCCACATATGTTGTTGGTGTCAATGCTGATCTCTACAACCCTGATGAATCTATCATC
AGCAATGCCTCTTGCACCACCAACTGCCTTGCTCCTTTTGTCAAGGTTCTTGACCAAAAATTCGG
CATTATCAAGGGAACCATGACAACTACTCACTCTTACACTGGTGACCAAAGGCTTCTTGATGCG
AGCCACAGGGACCTTAGACGTGCACGAGCTGCAGCCCTCAACATTGTTCCAACCTCAACTGGT
GCTGCTAAGGCCGTGGCCCTTGTCCTCCCAAGCCTTAAGGGGAAGCTCAACGGCATTGCCCTCC
GTGTGCCAACCCCTAACGTCTCGGTTGTTGACCTTGTCGTTCAAGTCTCCAAGAAGACATTTGCT
GAGGAAGTGAATGCTGCATTTAGGGAGGCTGCTGACAAGGAGCTCAAGGGAATTCTCGATGTC
TGCGATGAACCACTCGTGTCAGTCGACTTTCGGTGCAGTGATGTGTCATCAACTGTTGATGCTTC
ACTTACTATGGTCATGGGAGATGACATGGTTAAGGTTATTGCTTGGTATGACAATGAATGGGGTT
ACTCACAGAGGGTGGTTGATCTTGCTGACATTGTTGCAAACCAGTGGAAATAA

NbPsbQ1 (Niben101Scf06113g00021.1)
ATGGCTCATGCTATGGCTTCAATGGGTGGCCTAATTGGTTCTTCTCAAACTGTGTTGGATGGTCA
GCTCTGTGGTTCAGCCCGTTTGAGCACTGTTAGCACCAGCAGAATTGCCTTGGCTAGACCAGGT
CTCAGCATTAGAGCCCAACAGGGGTCTGCTGACACTGAAACTAGCCGTAGAGCCGTCATCGGT

CTTGTTGCTGCTGGCCTTGCTGGTTCCTTTGCTCAAGCAGCCTTTGCTGCAGCTAAATCAATCAA
GATTGGTGGCGCTCCTCCTCCCTCCGGTGGATTACCTGGAACTTTGAACTCGGATGAGGCAAGG
GACTTTGGTCTACCACTTAAGAAGAGGTTTTACCTTCAACCATTGACTCCAGCTGAAGCAGCCC
AGAGAGTTAAGGATTCAGCCAAGGAGATTGTTAGCGTCAAGAATTTCATCGACAAGAAGGCCT
GGCCATATGTCCAGAATGACCTTCGTCTCAGAGCAGAATACCTTCGCTATGACCTTAAAACCGT
AATCTCAGCTAAGCCAAAAGAAGAGAAGGGAAAGCTCCAGGACCTGACTGGAAAGCTCTTCAA
GACCATTAGTGATCTGGACCATGCAGCAAAGACCAAGAACAGCCCTGAAGCAGAGAAGTACTA
TGCTGAAACTGTATCTACCTTAAATGATGTTTTGGCCAAACTTGGTTAA
